# Mediterranean diet adherence and tirzepatide: real-world evidence on adiposity indices and insulin resistance beyond weight loss

**DOI:** 10.3389/fendo.2025.1700894

**Published:** 2026-01-14

**Authors:** Valentina Paternò, Giulio Geraci, Tommaso Piticchio, Rosario Le Moli, Stefano Burgio, Gabriele Costanzo, Gianluca Sambataro, Roberto Baratta, Federica Barbagallo, Francesco Pallotti

**Affiliations:** 1 Internal Medicine Unit, Hospital “Umberto I”, Azienda Sanitaria Provinciale di Enna, Enna, Italy; 2 Department of Medicine and Surgery, University of Enna “Kore”, Enna, Italy; 3 Azienda Sanitaria Provinciale di Catania, Catania, Italy; 4 Endocrinology Unit, ARNAS (Azienda di Rilievo Nazionale e di Alta Specializzazione – National High-Specialty Healthcare Trust) Garibaldi Hospital, Catania, Italy; 5 Diabetology Unit, Hospital “Umberto I”, Azienda Sanitaria Provinciale di Enna, Enna, Italy

**Keywords:** obesity, weight loss, adiposity index, VAI, tirzepatide

## Abstract

**Introduction:**

Tirzepatide, a dual GIP/GLP-1 receptor agonist, is highly effective in reducing body weight and improving glucometabolic outcomes. However, most clinical trials have overlooked the role of diet quality, leaving unclear whether lifestyle factors may modulate pharmacological benefits. The Mediterranean diet, assessed through the validated PREDIMED score, has proven effects on visceral adiposity and metabolic health and may act synergistically with novel incretin therapies.

**Materials and methods:**

We enrolled 53 patients with overweight/obesity eligible for tirzepatide (BMI ≥ 30 kg/m^2^ or BMI ≥ 27 kg/m^2^ with comorbidities). Participants were clinically assessed at baseline (T0) and after a median of 3 months (T1) of treatment (2.5 mg/week for 1 month, then 5.0 mg/week). All patients received personalized recommendations for a Mediterranean dietary plan. Anthropometric measures, adiposity indices (BMI, WC, WtHR, BRI, ABSI, VAI), biochemical parameters, and PREDIMED scores were collected at both visits. Correlations and generalized linear models were applied to explore the relationship between dietary adherence and changes in adiposity indices.

**Results:**

After three months, patients showed significant reductions in weight, BMI, WC, WtHR, BRI, and VAI (all p < 0.05), while ABSI remained unchanged. The glucometabolic profile improved consistently, with declines in fasting glucose, insulin, HbA1c, and triglycerides, alongside higher HDL-c. PREDIMED scores increased substantially (mean +3.2 points, p < 0.001). Higher adherence to the Mediterranean diet was associated with lower insulin levels, improved HOMA index, and greater reductions in VAI, even after adjusting for age, gender, and BMI.

**Conclusion:**

This real-world study confirms the efficacy of tirzepatide on adiposity and metabolic markers and provides exploratory evidence that adherence to a Mediterranean diet enhances its impact on visceral adiposity. The combination of pharmacological therapy and diet quality may offer additive benefits, and the integration of both PREDIMED and VAI in future studies could support more comprehensive strategies for cardiometabolic risk stratification and obesity care.

## 1 Introduction

Obesity represents a major global health challenge, strongly associated with type 2 diabetes, cardiovascular disease, liver steatosis, and premature mortality. In recent years, incretin-based treatments have recently reshaped obesity management. Glucagon-like peptide-1 receptor agonists (GLP-1RAs) and, more recently, the dual GIP/GLP-1 receptor agonist tirzepatide, have demonstrated remarkable efficacy in reducing body weight and improving glycemic and cardiometabolic profiles, placing them at the center of obesity care (1). Despite this pharmacological progress, the role of diet quality, a cornerstone of obesity care, has received limited attention. Evidence supporting the Mediterranean diet is extensive, yet most trials involving incretin therapies rarely report dietary adherence. A recent review found that fewer than 10% of incretin trials provided meaningful dietary data making it difficult to disentangle the relative contributions of pharmacologic and lifestyle factors to observed metabolic improvements (2). The Mediterranean diet, characterized by abundant consumption of vegetables, fruits, legumes, whole grains, fish, and extra-virgin olive oil, alongside a low intake of red meat and ultra-processed foods, is consistently associated with improved insulin sensitivity, lower cardiovascular risk, reduced visceral adiposity and markers of hepatic steatosis (3–5). Integrating this dietary model with modern incretin therapies could enhance metabolic outcomes through complementary mechanisms. In parallel, increasing attention has been given to precise measures of body composition. Traditional metrics such as Body Mass Index (BMI), while useful at a population level, fail to capture fat distribution and metabolic risk which is strictly linked to central adiposity. Alternative indices including waist circumference (WC), waist-to-height ratio (WtHR), body roundness index (BRI), a body shape index (ABSI), and the visceral adiposity index (VAI) together with laboratory measures such as fasting glucose, insulin, HbA1c, lipid profile and liver enzymes, offer better assessment of central adiposity and its systemic impact (6–9). Adherence to the Mediterranean diet can be reliably assessed using a validated tool, the 14-item PREDIMED score, which correlates with improved cardiometabolic profiles and long-term prognosis (10). However, the potential interaction between tirzepatide efficacy and Mediterranean diet adherence has not yet been systematically explored (2). Therefore, this real-world study aimed to evaluate whether adherence to the Mediterranean diet, assessed by the PREDIMED score, influences changes in adiposity indices (BMI, WC, WtHR, BRI, ABSI, VAI) and metabolic parameters (fasting glucose, insulin, HbA1c, lipid profile, and liver enzymes) in patients treated with tirzepatide. Specifically, we investigated whether improvements in diet quality during pharmacological treatment confers additional benefits on visceral adiposity and metabolic health, thereby supporting the development of integrated diet–drug models of care.

## 2 Materials and methods

### 2.1 Study design and population

The study was approved by the Local Ethics Committee (Catania 2, protocol n. 116/CEL, 21 May 2024), and all participants provided written informed consent. This prospective real-world study was conducted between January and July 2025 at the Endocrinology and Diabetology Outpatient Clinic of Umberto I Hospital, Enna. A total of 53 adult patients with overweight or obesity were enrolled among those attending the clinic for metabolic assessment and who were eligible for tirzepatide treatment. Participants were clinically evaluated at baseline (T0) and after a median of 3 months (T1) of therapy. Tirzepatide was administered at 2.5 mg/week for the first month and 5.0 mg/week thereafter, according to standard clinical practice. During the treatment period, all patients were advised to follow a personalized Mediterranean diet plan, tailored to individual caloric requirements and discussed with a trained clinician at each visit. The main objective of the study was to evaluate adherence to the Mediterranean diet before and after tirzepatide treatment and to determine whether higher adherence was associated with improvements in adiposity indices and metabolic parameters. Inclusion criteria were: 1) adults with overweight or obesity and eligibility for tirzepatide treatment according to current clinical indications (BMI ≥ 30 kg/m², or ≥ 27 kg/m² with obesity-related comorbidities); 2) compliance with the prescribed Mediterranean diet plan and follow-up schedule; 3) adherence to the prescribed pharmacological treatment. Exclusion criteria included: previous or current treatment with other GLP-1 receptor agonists; active malignancy; known eating or nutritional disorders; use of psychiatric and other medications potentially affecting appetite or metabolism. At each visit, routine biochemical assessments (including complete blood count, glucometabolic profile, and renal and hepatic function) and clinical parameters (body weight, height, and waist circumference) were collected, and the PREDIMED questionnaire was administered to evaluate adherence to the Mediterranean diet. Height and body weight were measured using a mechanical scale with integrated stadiometer (SECA GmbH & Co. KG, Hamburg, Germany), with participants wearing light clothing and no shoes. Blood samples were analyzed in the central laboratory of our hospital according to standardized protocols.

### 2.2 Sample size and sensitivity analysis

Given the exploratory design, a sample size calculation was performed only for the primary dietary endpoint (change in PREDIMED score), while a *post hoc* sensitivity analysis was conducted for the VAI endpoint. The sample size for PREDIMED was calculated assuming a minimal clinically important difference (MCID) of 2 points on the 14-item score, a within-subject standard deviation (SD) of 3–4, a pre–post correlation of 0.5, a two-tailed α = 0.05, and a power of 0.80, leading to a required number of participants of 32. The final sample (n = 53) therefore provided sufficient statistical power to detect clinically meaningful improvements in Mediterranean diet adherence. For VAI, no prior data were available to estimate the expected effect of tirzepatide; thus, a formal *a priori* calculation was not feasible. A *post hoc* sensitivity analysis was instead conducted and indicated that with n = 53, α = 0.05 (two-tailed), and a paired design, the study had approximately 80% power to detect a within-subject standardized effect size of d^z^ ≈ 0.40 (medium effect).

### 2.3 Statistical analysis

Continuous variables are presented as mean ± SD or as median and interquartile range, as appropriate. Normality of distributions was tested using the Kolmogorov-Smirnov test. Either Paired T test or Wilcoxon signed rank test have been used to compare baseline T0 vs follow up T1 distributions. Categorical variables are presented as counts and/or percentages and differences in frequencies are performed by Fisher’s exact test. The presence of statistically significant correlations among considered parameters was evaluated using Spearman’s rank correlation test. Finally, associations between adiposity indices, PREDIMED score, age, gender and other relevant variables have been investigated by uni- and multivariate generalized linear models. A two-tailed P-value ≤ 0.05 was considered significant. All computations were carried out with the software R version 4.4.1 (2024 The R Foundation for Statistical Computing, Vienna, Austria. URL https://www.R-project.org/).

## 3 Results

Among 335 individuals attending our outpatient clinic for metabolic assessment during the recruitment window, 137 patients met the preliminary clinical criteria for potential eligibility for tirzepatide therapy and were formally screened. Of these, 84 subjects were excluded for the following reasons: previous GLP-1RA treatment (n = 36), psychiatric medication use (n = 16), or unwillingness to provide informed consent and/or complete follow-up assessments (n = 32), while 53 subjects fulfilled all inclusion criteria, agreed to participate, and were enrolled. (**Supplementary Figure 1**). These 53 patients completed baseline (T0) and follow-up (T1) assessments after a median of 3.2 months (range 2–5 months) of treatment between January and July 2025. Among those subjects 32 (60.4%) were women and 21 (39.6%) were men. **Supplementary Table S1** reports baseline clinical characteristics and prevalence of major comorbidities in the study cohort. No significant adverse events were reported, and no patient has discontinued the treatment.

*Glucometabolic profile***-****Table 1** reports paired comparisons of glucometabolic parameters at baseline (T0) and follow up. In particular, fasting glucose declined from 118.5 ± 64.4 to 91.3 ± 15.8 mg/dL (p < 0.001) accompanied by significant reductions in fasting insulin (24.7 ± 16.4 vs. 17.8 ± 9.7 µU/mL; p < 0.001) and HbA1c (6.4 ± 1.5% vs. 5.7 ± 0.6%; p < 0.001). Liver enzymes significantly improved (all p ≤ 0.020). On the other hand, kidney function parameters (creatinine and eGFR) did not change significantly after treatment. Lipid profile showed favorable changes, with a significant but clinically modest reduction in total cholesterol (193.8 ± 34.8 vs. 184.2 ± 27.1 mg/dL; p = 0.020), a significant increase in HDL-c (45.4 ± 8.0 vs. 46.9 ± 7.5 mg/dL; p = 0.006), and decreases in LDL-c (116.4 ± 26.3 vs. 108.6 ± 20.7 mg/dL; p < 0.001) and triglycerides (158.9 ± 62.3 vs. 135.9 ± 52.6 mg/dL; p = 0.002).

**Table 1:** Glycometabolic parameters at baseline (T0) and post treatment (T1).

Parameter	Baseline (T0)	Post treatment (T1)	p-value
Glucose (mg/dl)	118.5 ± 64.4 101.0 (88.0 – 116.0)	91.3 ± 15.8 86.5 (83.0 - 98.0)	<0.001
Fasting Insulin (UI/ml)	24.7 ± 16.4 19.0 (13.6 - 32.6)	17.8 ± 9.7 15.8 (10.7 - 25.0)	<0.001
HbA1c (%)	6.4 ± 1.5 6.0 (5.8 - 6.4)	5.7 ± 0.6 5.6 (5.3 – 5.9)	<0.001
Creatinine (mg/dl)	0.9 ± 0.2 0.9 (0.8 - 1.1)	0.9 ± 0.2 0.9 (0.8 - 1.0)	0.235
eGFR	89.9 ± 20.4 90.9 (75.4 - 105.8)	92.2 ± 20.6 95.8 (79.2 - 108.7)	0.251
AST	30.8 ± 16.3 24.0 (20.0 - 42.0)	25.1 ± 10.3 22.0 (17.0 - 31.0)	<0.001
ALT	33.8 ± 17.0 28.0 (21.0 - 44.0)	25.0 ± 9.7 22.5 (18.0 - 34.0)	<0.001
γGT	38.1 ± 18.4 35.0 (22.0 - 50.0)	29.6 ± 13.4 24.5 (19.0 - 40.0)	0.020
Total Cholesterol (mg/dl)	193.8 ± 34.8 200.0 (171.6 - 215.0)	184.2 ± 27.1 182.5 (170.0 - 205.0)	0.020
HDL-c (mg/dl)	45.4 ± 8.0, 46.0 (39.0, 51.0)	46.9 ± 7.5, 47.0 (41.0, 54.0)	0.006
LDL-c (mg/dl)	116.4 ± 26.3, 116.0 (100.0, 136.0)	108.6 ± 20.7, 105.5 (95.0, 125.0)	<0.001
Triglycerides (mg/dl)	158.9 ± 62.3, 149.5 (114.0, 176.5)	135.9 ± 52.6, 124.0 (100.0, 167.0)	0.002

Wilcoxon signed rank test. Data are presented as mean ± SD, and Median (Q1, Q3).

*Anthropometric and adiposity indices* – **Table 2** shows main changes in anthropometric measures and adiposity indices. Mean body weight decreased from 110.3 ± 20.5 kg to 99.2 ± 18.9 kg (p = 0.002), with a parallel reduction in BMI (39.8 ± 5.8 vs. 35.5 ± 5.6 kg/m²; p < 0.001) and WC (122.9 ± 16.9 vs. 113.4 ± 15.8 cm; p < 0.001). Composite indices of central adiposity showed consistent trends, with a significant reduction in WtHR (0.74 ± 0.09 vs. 0.68 ± 0.09; p < 0.001) and BRI (9.08 ± 2.78 vs. 7.44 ± 2.43; p < 0.001), while ABSI remained unchanged. VAI decreased significantly (6.32 ± 2.76 vs. 5.06 ± 2.01; p = 0.015). As expected, BMI correlated positively with fasting glucose (ρ = 0.424, *p* = 0.003), HbA1c (ρ = 0.344, *p* = 0.018), and insulin (ρ = 0.327, *p* = 0.026), while WC showed strong positive correlations with fasting glucose (ρ = 0.459, *p* = 0.001), ALT (ρ = 0.483, *p* < 0.001), and triglycerides (ρ = 0.301, *p* = 0.040). Similar patterns were noted for WtHR and BRI, both associated with fasting glucose (ρ = 0.375, *p* = 0.009 for both) and triglycerides (ρ = 0.318, *p* = 0.029 and ρ = 0.291, p = 0.048, respectively). ABSI showed only a correlation trend with HDL-c (ρ = -0.278, p = 0.070) (**Supplementary Table 2**).

**Table 2:** Baseline (T0) and post treatment (T1) anthropometric measures, adiposity/metabolic indices, and PREDIMED score.

Parameter	Baseline (T0)	Post treatment (T1)	p-value
Age (years)	44.4 ± 11.8 43.0 (37.0 - 53.0)	N/A	N/A
Weight (Kg)	110.3 ± 20.5 111.0 (98.0 - 120.0)	99.2 ± 18.9 99.5 (86.2 – 107.5)	0.002
Height (m)	1.67 ± 0.09 1.67 (1.60 - 1.74)	N/A	N/A
BMI (Kg/m^2^)	39.8 ± 5.8 38.8 (35.7 - 42.9)	35.5 ± 5.6 34.5 (31.5 - 38.8)	<0.001
WC (cm)	122.96 ± 16.89, 121.50 (109.50, 132.00)	113.45 ± 15.82, 112.35 (103.00, 123.00)	<0.001
WtHR	0.74 ± 0.09 0.72 (0.67 - 0.79)	0.68 ± 0.09 0.67 (0.60 - 0.73)	<0.001
ABSI	0.82 ± 0.07, 0.82 (0.77, 0.86)	0.81 ± 0.08, 0.80 (0.76, 0.86)	0.329
BRI	9.08 ± 2.78, 8.54 (7.02 - 10.57)	7.44 ± 2.43 7.00 (5.56 - 8.82)	<0.001
PREDIMED	6.30 ± 1.89 6.00 (5.00 - 7.00)	9.52 ± 2.34 9.00 (8.00 - 11.00)	<0.001
VAI	6.32 ± 2.76 5.82 (4.75 - 7.20)	5.06 ± 2.01 4.93 (3.543 - 5.93)	0.015

Wilcoxon signed rank test. Results are presented as mean ± SD and Median (Q1, Q3).

**Figure 1:**
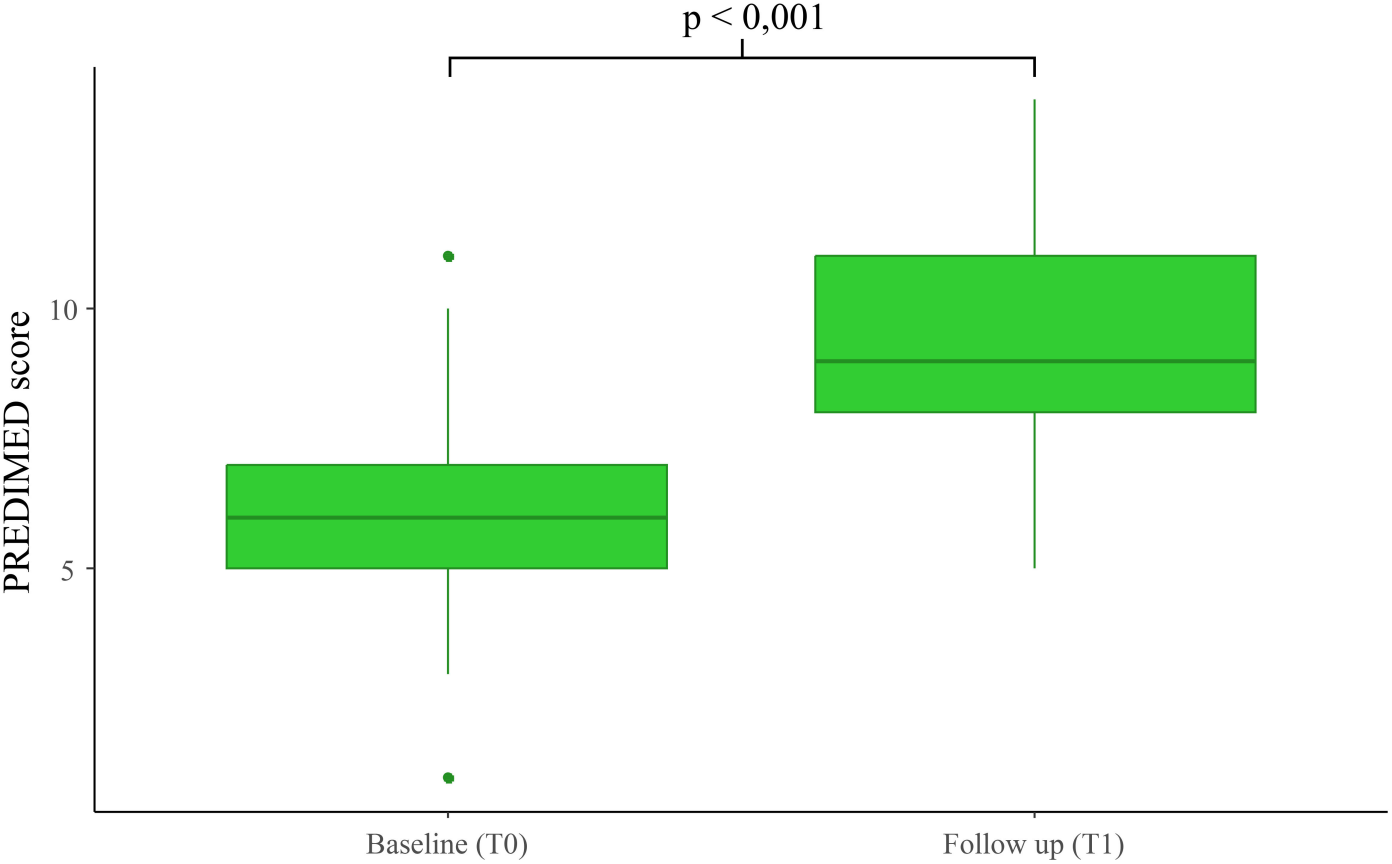
Boxplot of PREDIMED score before at baseline (T0) and at follow-up (T1).

*PREDIMED score -* Adherence to the Mediterranean diet, assessed through the PREDIMED score, increased substantially (6.30 ± 1.89 vs. 9.52 ± 2.34; p < 0.001) (**Table 2**, **Figure 1**). **Table 3** shows relevant correlations with PREDIMED score. In brief, a higher adherence to the Mediterranean diet (as reflected by higher PREDIMED scores) showed significant correlations with lower fasting insulin levels (ρ = –0.294, *p* = 0.033) that further improved post treatment with Tirzepatide (ρ = –0.402, *p* = 0.006) with similar correlations with the HOMA index. Moreover, PREDIMED score negatively correlates with lower triglycerides (ρ = –0.343, *p* = 0.024) only after treatment, suggesting a more favorable metabolic profile in individuals with greater diet quality. Although no significant associations were detected with the improvements of the adiposity indices considered (**Table 3d**), our exploratory analyses detected a significant correlation with ΔVAI (ρ = –0.354, *p* = 0.021).

Table 3a–dSpearman correlation coefficients for PREDIMED scores and other clinical variables and adiposity indices.

**Table:** 

Table 3a	Coefficient	Weight (kg)	BMI (Kg/m^2^)	WC CV (cm)	WtHR	ABSI	BRI	VAI
PREDIMED (Baseline T0)	Spearman Rho	-0,214	-0,088	0,026	0,085	0,144	0,077	0,179
	p-value	0,125	0,529	0,854	0,545	0,303	0,584	0,228
PREDIMED (Follow up T1)	Spearman Rho	-0,113	-0,122	0,046	0,092	0,216	0,056	-0,296
	p-value	0,438	0,404	0,755	0,531	0,136	0,705	0,054

**Table:** 

Table 3b	Coefficient	Fasting Glucose	Fasting Insulin	HOMA index	HbA1c (%)	AST	ALT	γGT	Creatinine	eGFR
PREDIMED (Baseline T0)	Spearman Rho	-0,168	-0,294	-0,294	-0,177	0,116	0,05	-0,133	-0,086	-0,127
	p-value	0,23	0,033	0,032	0,204	0,406	0,721	0,341	0,545	0,371
PREDIMED (Follow up T1)	Spearman Rho	-0,208	-0,402	-0,429	-0,086	0,14	0,155	0,093	0,068	-0,247
	p-value	0,16	0,006	0,003	0,567	0,355	0,304	0,536	0,647	0,094

**Table:** 

Table 3c	Coefficient	Total Cholesterol	HDL-c	LDL-c	Triglycerides
PREDIMED (Baseline T0)	Spearman Rho	0,006	-0,085	0,109	-0,032
	p-value	0,97	0,569	0,445	0,827
PREDIMED (Follow up T1)	Spearman Rho	-0,177	0,142	0,059	-0,343
	p-value	0,256	0,364	0,696	0,024

**Table:** 

Table 3d	Coefficient	ΔWeight	ΔBMI	ΔCV	ΔWtHR	ΔBRI	ΔABSI	ΔHOMA	ΔVAI
PREDIMED (Follow up T1)	Spearman Rho	0,045	0,003	0,08	0,124	0,027	0,069	0,253	-0,354
	p-value	0,757	0,986	0,583	0,396	0,855	0,638	0,096	0,021

**Figure 2:**
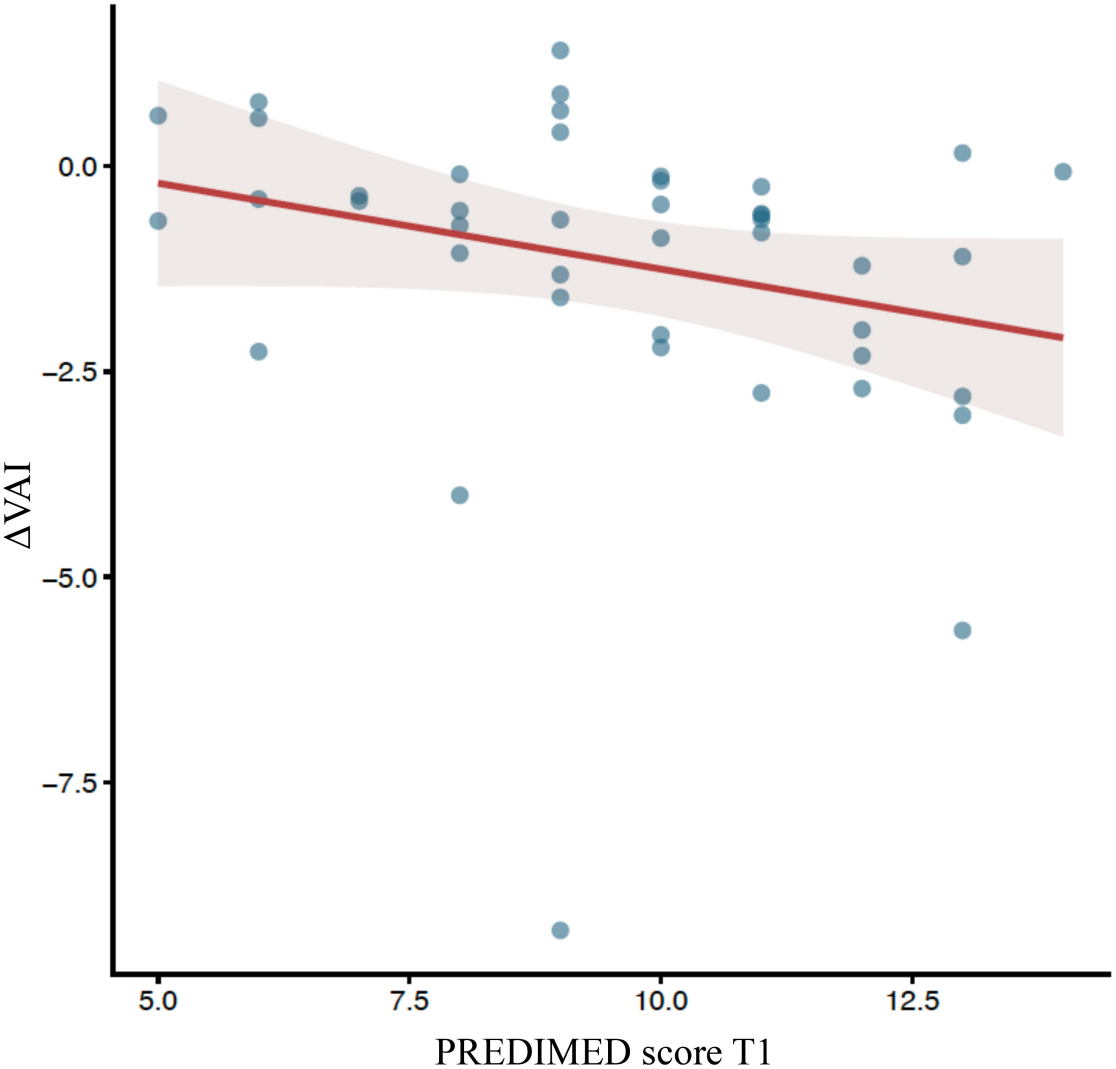
Scatterplot of PREDIMED at baseline (T0) and at follow-up (T1) and ΔVAI.

*Linear models -* To further explore predictors of adiposity index reduction, we constructed multivariable linear models including ΔBMI, ΔWtHR, ΔBRI and ΔABSI as dependent variables, with age, PREDIMED score and gender as independent variable (**Tables 4a–d**). For all these indices, no significant association with PREDIMED score was detected. For ΔBMI, higher age was significantly associated with greater BMI reduction (β = 0.308, p = 0.050), independently from adherence to mediterranean diet and gender, which did not reach statistical significance. All these observations suggest that mediterranean diet adherence did not impact significantly to these indices during Tirzepatide treatment. However, we observed that ΔVAI was significantly associated with final PREDIMED score (**Figure 2**), as higher values predicted a greater reduction in VAI, even when adjusting for age, gender and BMI (β = -0.383, p = 0.037) or ΔBMI (β = -0.304, p = 0.042). This last observation suggests a potential beneficial role of Mediterranean diet adherence in modulating visceral adiposity changes during Tirzepatide treatment. Finally, to investigate whether Tirzepatide was the main determinant of Mediterranean diet adherence, we performed an additional multivariable linear model using ΔPREDIMED as dependent variable and including ΔBMI (proxy of tirzepatide pharmacological effect), age, gender, baseline PREDIMED, baseline BMI as independent variables. ΔBMI was not associated with ΔPREDIMED (β = –0.053, p = 0.746), indicating that improvement in diet adherence was not driven by weight loss or by the pharmacological effect of tirzepatide, while age was positively associated with ΔPREDIMED (β = +0.055, p = 0.040) and Baseline PREDIMED was inversely associated with ΔPREDIMED (β = –0.368, p = 0.022), consistent with a ceiling effect. Gender and baseline BMI, instead, were not significant predictors (**Table 5**).

**Table 4:** Linear models investigating associations of BMI (1a), WtHR (1b), BRI (1c) and VAI (1c) reduction.

Table 4a – Dependent variable ΔBMI				
Effect	Estimate	SE	β	p-value
Age	0.050	0.025	0.308	0.050
PREDIMED score	-0.047	0.123	-0.058	0.702
GENDER (Male – Female)	-0.992	0.566	-0.527	0.086

Table 4b – Dependent variable ΔWtHR				
Effect	Estimate	SE	β	p-value
ETA	-0.001	0.001	-0.042	0.795
PREDIMED score	0.003	0.002	0.178	0.267
GENDER (Male – Female)	0.004	0.009	0.126	0.691

Table 4c – Dependent variable ΔBRI				
Effect	Estimate	SE	β	p-value
Age	-0.014	0.012	-0.1895	0.241
PREDIMED score	0.035	0.058	0.09	0.551
GENDER (Male – Female)	-0.017	0.267	-0.020	0.950

Table 4d – Dependent variable ΔABSI				
Effect	Estimate	SE	β	p-value
Age	-0.001	0.001	-0.266	0.095
PREDIMED score	0.002	0.002	0.159	0.309
GENDER (Male – Female)	0.011	0.009	0.364	0.243

**Table 5:** Linear models investigating associations of ΔPREDIMED score.

Table 5 – Dependent variable ΔPREDIMED				
Effect	Estimate	SE	β	p-value
ΔBMI	-0.053	0.163	0.948	0.745
Age	0.055	0.026	1.056	0.040
GENDER (Male – Female)	-0.168	0.642	0.845	0.794
PREDIMED (Baseline T0)	-0.367	0.155	0.692	0.022
BMI (Baseline T0)	-0.035	0.049	0.965	0.474

## 4 Discussion

Our data provides evidence from a real-world cohort on the complex interplay between tirzepatide, diet quality, and adiposity indices. First, a relevant methodological aspect concerns the dose of tirzepatide used. The 5 mg/week dose was chosen to reflect real-world clinical practice and to minimize gastrointestinal side effects during the initial treatment phase. We followed the standard titration scheme (2.5 mg/week for 4 weeks, then 5 mg/week), which represents the most commonly prescribed early maintenance dose in obesity management. Large clinical trials and their *post hoc* analyses have already established the efficacy of tirzepatide in reducing weight, visceral fat, and enhancing glycemic control, ultimately improving cardiometabolic outcomes (11–13). Although emerging evidence on specific nutritional approaches (e.g., ketogenic or low-energy diets) indicate that they may potentiate hepatic and metabolic benefits beyond weight loss (14) and even reduce inflammation and oxidative stress synergistically with other drugs (15) nutritional aspects are somewhat underreported in recent studies. Our data show that PREDIMED scores significantly improved after a median of about 3 months of Tirzepatide treatment. The improvement in PREDIMED score was not associated with the magnitude of weight loss, indicating that tirzepatide did not directly drive the observed change in diet quality. Rather, higher age and low baseline adherence emerged as significant predictors of improvement, consistent with a ceiling effect and with greater dietary engagement in older adults. In fact, at baseline, all participants received a brief educational session with a trained clinician on the principles of the Mediterranean diet, including food selection and portion guidance. Improvements in PREDIMED scores likely reflect both lifestyle counseling and treatment-related changes in appetite and food choices. This observation slightly differs from a recent study where dietary educational session improves dietary knowledge but does not modify short-term (one month) adherence to the Mediterranean diet. This disagreement may reflect the use of different tools (PREDIMED vs MDAAQ) and follow-up duration (one vs three months) (16); however studies differ also by the concurrent use of tirzepatide in our cohort, which may facilitate diet adherence via appetite modulation potentially by impacting ingestive behavior (17). In this perspective, pharmacotherapy could act as a modulator, increasing the effect of brief counseling and translating into measurable improvements in diet quality. We should also remark that our study highlights the clinical usefulness of adiposity indices for early follow up of overweight/obese subjects. In particular, while BMI and BRI were significantly reduced after tirzepatide treatment, ABSI remained unchanged. This could reflect the different nature of these indices: while BMI and BRI are sensitive to short-term weight and waist changes, ABSI is primarily a marker of body shape and mortality risk and could be less responsive to short-term interventions. Thus, ABSI may be less responsive to short-term pharmacological interventions, and that indices such as VAI or BRI could provide more clinically relevant information when assessing early changes in adiposity. Thus, by combining the PREDIMED score with longitudinal measures of adiposity such as VAI, WtHR, and BRI, our study brings preliminary exploratory data to address this limitation. We observed a clear reduction in weight, waist circumference, and VAI after three months of therapy, and patients with higher PREDIMED scores showed more favorable metabolic profiles. The significant improvement of almost all adiposity indices and glucometabolic profile markers under Tirzepatide treatment, clearly confirms recent literature (11) and its possible use as a further treatment strategy in consolidated primary and secondary cardiovascular prevention strategies (18). These findings are also consistent with previous evidence showing that incretin-based therapies improve visceral adiposity indices. In women with PCOS, the addition of semaglutide to metformin led to a pronounced reduction in BMI, waist circumference, and cVAI. These results confirm that incretin therapy can also impact visceral fat and metabolic inflammation, in line with our observation of reduced VAI under tirzepatide treatment (19). Mediterranean diet effects on visceral adiposity indices and metabolic parameters are known, as demonstrated in a recent systematic review and meta-analysis of RCTs evaluating lifestyle interventions in MASLD. In fact, Arita et al. demonstrated that the Mediterranean diet consistently reduced body weight, BMI, WC, and ALT levels, while also showing beneficial effects on steatosis and, in some trials, fibrosis (20). Another interesting aspect is the compliance to Mediterranean diet regimen, which appears strongly associated with cardiovascular and metabolic risk reduction (21–23), as low adherence doubles the odds of cardiovascular disease in patients with metabolic syndrome. As shown by the ATTICA study, a 10% increase in adherence to the Mediterranean Diet is linked to a 15% reduction in the odds of developing cardiovascular disease (CVD) and reduces the effect of the single components of metabolic syndrome like waist circumference, LDL-c and hypertension on cardiovascular risk (24). We could not detect significant associations between PREDIMED and most adiposity indices like BRI and ABSI, likely because of the relatively limited sample size, causing Tirzepatide to hide the expected effects of the Mediterranean diet adherence. Nonetheless, the T1 PREDIMED score was associated with a greater reduction in VAI, even after adjusting for confounders (age, gender, BMI), further highlighting the additional beneficial role of the Mediterranean diet adherence during Tirzepatide treatment. The VAI is a composite measure that captures both body fat distribution and lipid profile, offering a practical proxy that summarizes function and distribution of adipose tissue, which has been demonstrated to predict the risk of cardiometabolic disease (25). As highlighted in a recent review, VAI has strong associations with insulin resistance, metabolic syndrome, hepatic steatosis, and cardiovascular disease (26) and it has been shown to be a good index to identify adolescents at risk of metabolic syndrome (27). In our study, the association of PREDIMED and VAI remarks that the achieved reductions in visceral fat are strongly linked with metabolic improvements, as shown by a recent dietary intervention, where a ≥5% decrease in visceral fat area translated into significant benefits in lipid profile and glycemic control (28). This reinforces the notion that the Mediterranean diet, when combined with pharmacological therapy, may enhance the effects on visceral fat reduction and metabolic benefits. Our results align with population-based evidence linking new adiposity indices, including VAI, to the cardiovascular–kidney–metabolic (CKM) continuum. Improving diet quality may thus potentiate pharmacological effects on visceral fat, offering a strategy to mitigate CKM risk (29–31). Nutritional factors also play a role, with biomarkers such as advanced glycation end products linking poor diet quality to CKM progression (31). Following adherence to MD protocol reduces low grade chronic inflammation, this effect is beyond the content in macronutrients of MD and it is related to MD bioactive compounds that can modulate the machinery of systemic inflammation (32). This reinforces the rationale for integrated diet–drug approaches aimed at reducing CKM risk in patients with obesity and diabetes. Real-world data have shown that both GLP-1 receptor agonists and structured dietary approaches can improve metabolic health: in particular, a mediterranean diet (~1500 kcal/day for women and ~1800 kcal/day for men) and a very low calorie ketogenic diet (< 800 kcal/day), can both improve weight, visceral adiposity, and metabolic parameters, with adherence emerging as a critical determinant of success (33). Likewise, systematic reviews consistently report that higher adherence to the Mediterranean diet not only reduces BMI, metabolic syndrome risk, and markers of inflammation but also provides the added value of environmental sustainability (34). Our results support the concept that pharmacological and dietary strategies should not be considered mutually exclusive but rather complementary: greater VAI reduction under tirzepatide with high mediterranean diet adherence reinforces the hypothesis of an additive effect, where diet quality strengthens the pharmacological benefits on visceral adiposity and metabolic health. Our study presents several strengths and weaknesses. A major strength of this study is the integration of a validated measure of Mediterranean diet adherence (PREDIMED) with longitudinal assessment of multiple adiposity indices, including VAI, in a real-world clinical setting. This multidimensional approach provides novel insights into the interplay between diet quality, pharmacotherapy, and visceral adiposity, addressing a relevant gap in current incretin-based trials. Several limitations should also be acknowledged. First, the relatively small sample size and short follow-up period limit the generalizability of our findings and may have reduced the power to detect weaker associations, particularly for adiposity indices other than VAI. Second, observational design precludes causal inference, and residual confounding from unmeasured lifestyle or clinical factors cannot be excluded. Third, dietary adherence was assessed using the PREDIMED score, a validated but self-reported tool that may be subject to recall bias. Finally, the strong pharmacological effect of tirzepatide may have overshadowed the independent contribution of dietary adherence to some outcomes. Future larger and longer studies, ideally with objective biomarkers of diet and adiposity, are needed to confirm these exploratory observations. It should also be remarked that the use of imaging (e.g. DEXA, MRI, ultrasound, etc.) would have enriched the analysis in regards of the effects on body composition and steatosis. However, since this study was conducted in a real-world study, such methods were not available for all participants. To ensure homogeneity, we relied on validated anthropometric and metabolic indices (VAI, BRI, ABSI, WHtR) as indirect but reproducible markers of visceral adiposity. Despite these limitations, our study provides real-world evidence on the interplay between pharmacological and dietary interventions, offering novel insights into how tirzepatide and Mediterranean diet adherence may synergistically influence visceral adiposity and metabolic health.

## 5 Conclusions

In conclusion, our data confirms positive effects of tirzepatide on body weight reduction, adiposity indices and metabolic markers improvements. Moreover, we could also detect a significant improvement in Mediterranean diet adherence, as assessed by the PREDIMED score, emerged as a key determinant of metabolic benefits during tirzepatide therapy. Patients with higher PREDIMED scores showed greater reductions in visceral adiposity, underscoring the importance of diet quality in modulating pharmacological effects. Among adiposity indices, the VAI was particularly responsive, reflecting changes not only in body fat distribution but also in lipid profile. This reinforces its role as a complementary tool, alongside dietary assessment, for monitoring treatment response and stratifying cardiometabolic risk. Taken together, our findings support the concept of a diet–drug synergy, where adherence to the Mediterranean diet amplifies the benefits of incretin-based therapies. Future studies integrating PREDIMED and VAI into standardized protocols may provide a more comprehensive framework for personalized obesity and diabetes care.

## Data Availability

The raw data supporting the conclusions of this article will be made available by the authors, without undue reservation.
