# Supplementary figures and images for: Mediterranean diet adherence and tirzepatide: real-world evidence on adiposity indices and insulin resistance beyond weight loss
Source: Front Endocrinol (Lausanne). 2026 Jan 14;16:1700894. doi: 10.3389/fendo.2025.1700894 (PMC12846957; doi:10.3389/fendo.2025.1700894)

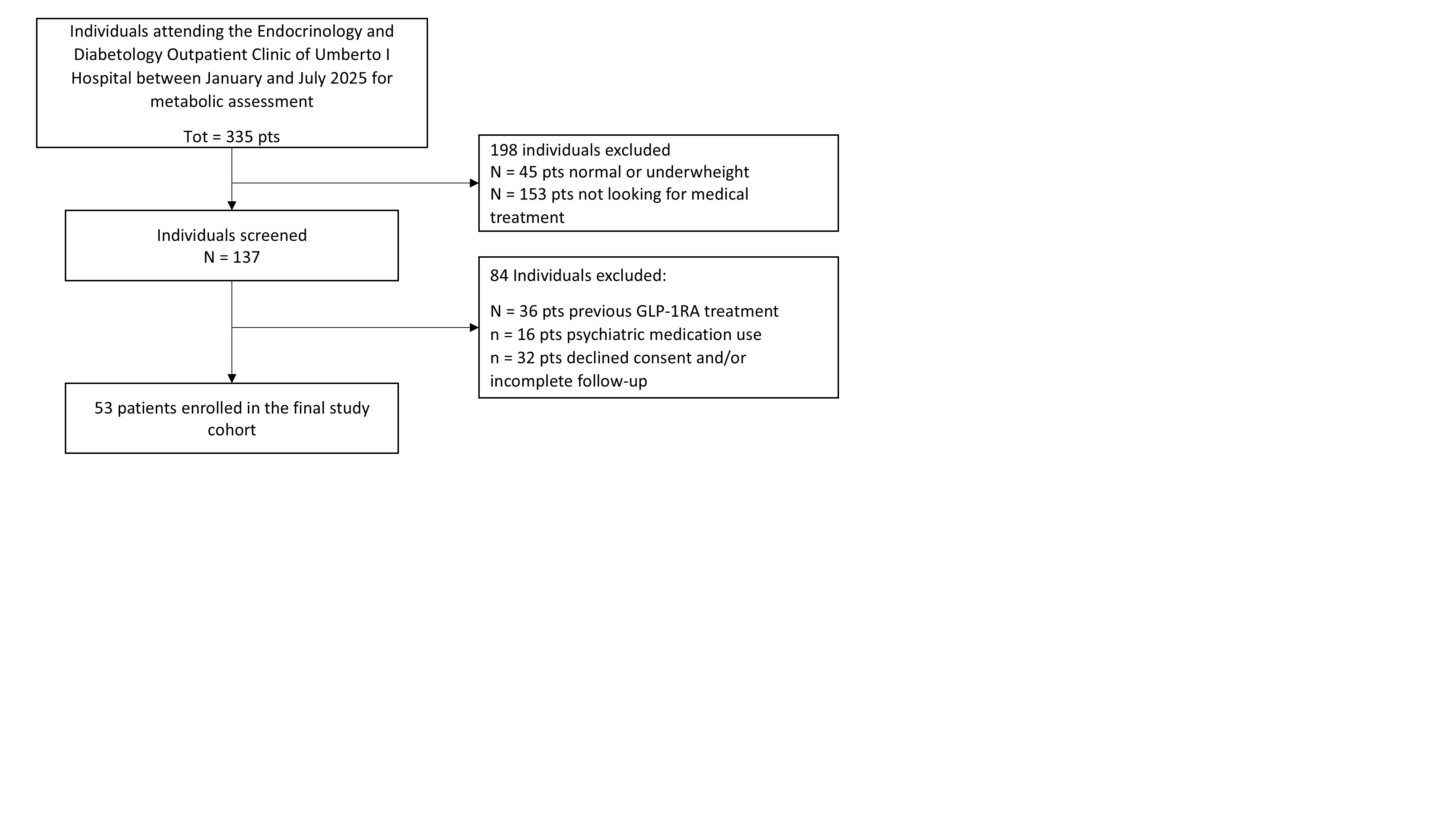

Supplement: Supplementary file 1 [file Image1.jpeg]
