# Supplementary material for: Mediterranean diet adherence and tirzepatide: real-world evidence on adiposity indices and insulin resistance beyond weight loss
Source: Front Endocrinol (Lausanne). 2026 Jan 14;16:1700894. doi: 10.3389/fendo.2025.1700894 (PMC12846957; doi:10.3389/fendo.2025.1700894)
Supplement: Supplementary file 2 [file Table1.docx]

| **Variable** | **Total (n = 53)** | **Women (n = 32)** | **Men (n = 21)** |
| --- | --- | --- | --- |
| **Smokers, n (%)** | 12 (22.6) | 7 (21.9) | 5 (23.8) |
| **Type 2 diabetes mellitus, n (%)** | 0 (0) | 0 (0) | 0 (0) |
| **Arterial hypertension, n (%)** | 22 (41.5) | 11 (34.4) | 11 (52.4) |
| **Dyslipidemia n (%)** | 20 (37.7) | 9 (28.1) | 11 (52.4) |
| **Hypothyroidism, n (%)*** | 9 (17.0) | 8 (25.0) | 1 (4.8) |
| **Polycystic ovary syndrome (PCOS), n (%)** | N/A | 6 (18.8) | N/A |
| **Current pharmacological treatment, n (%)** |  |  |  |
| **– Metformin** | 6 (11.3) | 4 (12.5) | 2 (9.5) |
| **– Statins** | 15 (28.3) | 9 (28.1) | 6 (28.6) |
| **– Antihypertensives** | 22 (41.5) | 11 (34.4) | 1. (52.4) |
| **– L-tiroxine*** | 9 (17.0) | 8 (25.0) | 1 (4.8) |

**Supplementary Table 1 –** Smoking status, prevalence of comorbidities and treatments in the study cohort. *all subjects were treated with L-Tiroxine and were euthytoid at enrollment.
