# Supplementary material for: Mediterranean diet adherence and tirzepatide: real-world evidence on adiposity indices and insulin resistance beyond weight loss
Source: Front Endocrinol (Lausanne). 2026 Jan 14;16:1700894. doi: 10.3389/fendo.2025.1700894 (PMC12846957; doi:10.3389/fendo.2025.1700894)
Supplement: Supplementary file 3 [file Table2.docx]

|  |  | **Fasting Glucose** | **HbA1c** | **Fasting Insulin** | **AST** | **ALT** | **GGT** | **Creatinine** | **eGFR** | **Total Cholesterol** | **HDL-c** | **LDL-c** | **Triglycerides** |
| --- | --- | --- | --- | --- | --- | --- | --- | --- | --- | --- | --- | --- | --- |
| **PREDIMED** | **Spearman Rho** | -0.208 | -0.086 | -0.402 | 0.140 | 0.155 | 0.093 | 0.068 | -0.247 | -0.177 | 0.142 | 0.059 | -0.343 |
|  | **p value** | 0.160 | 0.567 | 0.006 | 0.355 | 0.304 | 0.536 | 0.647 | 0.094 | 0.256 | 0.364 | 0.696 | 0.024 |
| **BMI** | **Spearman Rho** | 0.424 | 0.344 | 0.327 | 0.215 | 0.056 | 0.264 | 0.191 | -0.143 | 0.147 | 0.033 | 0.113 | 0.096 |
|  | **p value** | 0.003 | 0.018 | 0.026 | 0.152 | 0.712 | 0.073 | 0.199 | 0.339 | 0.346 | 0.831 | 0.449 | 0.539 |
| **WC** | **Spearman Rho** | 0.459 | 0.362 | 0.128 | 0.344 | 0.225 | 0.483 | 0.070 | -0.021 | 0.156 | -0.137 | 0.301 | 0.120 |
|  | **p value** | 0.001 | 0.012 | 0.397 | 0.019 | 0.134 | <.001 | 0.639 | 0.889 | 0.317 | 0.383 | 0.040 | 0.444 |
| **WtHR** | **Spearman Rho** | 0.375 | 0.338 | 0.076 | 0.245 | 0.166 | 0.390 | -0.054 | -0.003 | 0.149 | -0.110 | 0.318 | 0.104 |
|  | **p value** | 0.009 | 0.020 | 0.615 | 0.100 | 0.270 | 0.007 | 0.719 | 0.986 | 0.340 | 0.484 | 0.029 | 0.507 |
| **BRI** | **Spearman Rho** | 0.375 | 0.327 | 0.109 | 0.282 | 0.144 | 0.373 | -0.039 | -0.018 | 0.112 | -0.075 | 0.291 | 0.066 |
|  | **p value** | 0.009 | 0.025 | 0.472 | 0.057 | 0.339 | 0.010 | 0.793 | 0.902 | 0.476 | 0.632 | 0.048 | 0.675 |
| **ABSI** | **Spearman Rho** | 0.193 | 0.147 | -0.208 | 0.215 | 0.175 | 0.292 | -0.157 | 0.122 | -0.007 | -0.187 | 0.264 | -0.029 |
|  | **p value** | 0.193 | 0.324 | 0.165 | 0.151 | 0.243 | 0.047 | 0.290 | 0.415 | 0.966 | 0.229 | 0.073 | 0.854 |
| **VAI** | **Spearman Rho** | 0.039 | -0.054 | 0.149 | -0.096 | -0.111 | -0.172 | -0.311 | 0.395 | 0.329 | -0.344 | 0.217 | 0.812 |
|  | **p value** | 0.806 | 0.730 | 0.346 | 0.546 | 0.485 | 0.269 | 0.043 | 0.009 | 0.031 | 0.024 | 0.162 | <.001 |

**Supplementary Table 2** – Spearman correlation of PREDIMED score, anthropometric measures and adiposity indices with glycometabolic profile and liver and kidney function.
